# Walking to protect against cognitive decline: the role of APOE genotype and sex

**DOI:** 10.1186/s13293-026-00860-6

**Published:** 2026-02-21

**Authors:** Joel S. Burma, Caterina Rosano, John R. Best, Eleanor M. Simonsick, Teresa Liu-Ambrose, Cindy K. Barha

**Affiliations:** 1 Barha Brain Health Laboratory, Faculty of Kinesiology, University of Calgary, Calgary, AB Canada; 2 Hotchkiss Brain Institute, University of Calgary, Calgary, AB Canada; 3 Libin Cardiovascular Institute, University of Calgary, Calgary, AB Canada; 4 School of Public Health, University of Pittsburgh, Pittsburgh, PA USA; 5 Department of Gerontology, Simon Fraser University, Burnaby, BC Canada; 6 Intramural Research Program, National Institute on Aging, Baltimore, MD USA; 7 Aging, Mobility, and Cognitive Neuroscience Laboratory, Department of Physical Therapy, University of British Columbia, Vancouver, BC Canada; 8 Djavad Mowafaghian Centre for Brain Health, Faculty of Medicine, University of British Columbia, Vancouver, BC Canada; 9 Faculty of Kinesiology, University of Calgary, Calgary, AB Canada

**Keywords:** APOE ε4, Physical Activity, Cognitive Decline, Sex Differences, Walking

## Abstract

**Background:**

The apolipoprotein E (*APOE*) ε4 allele is a risk factor for late-onset Alzheimer’s disease; however, risk varies by sex and lifestyle. Regular physical activity is known to mitigate cognitive decline; whether the degree of benefit differs by *APOE* genotype, sex, and race remains unknown.

**Methods:**

Analyses utilized data from 2,985 participants in the Health, Aging, and Body Composition (HABC) cohort, comprising community-dwelling black and white older adults followed for 10 years. Cognitive performance was assessed multiple times across the 10 years using the Digit Symbol Substitution Test (DSST) for executive functions and processing speed and the Modified Mini-Mental State Examination (3MS) for global cognition. *APOE* genotypes were categorized into ε2, ε3, and ε4 groups. Annual self-reported walking time was used to quantify physical activity. Linear mixed models and latent growth curve modeling examined the interactions between *APOE* genotype, sex, and walking on cognitive trajectories with adjustments for race, study location, health score, age, education attained, and body mass index.

**Results:**

*APOE* ε4 carriers demonstrated steeper declines in both DSST and 3MS scores compared to ε3 carriers, irrespective of sex (all β<-0.13, all *p* < 0.004). *APOE* ε2 was protective longitudinally for 3MS in females only (β = 0.15, *p* < 0.002). Walking showed the strongest protective effect in *APOE* ε4 carriers for females and males in the rate of change of DSST and 3MS scores (all β > 0.27, all *p* < 0.044).

**Discussion:**

These findings underscore the importance of public messaging about the benefits of regular physical activity for retaining cognitive function especially for persons genetically at heightened risk.

**Supplementary Information:**

The online version contains supplementary material available at 10.1186/s13293-026-00860-6.

## Abstract (plain language summary)

As we age, it is common for thinking skills such as memory, attention, and processing speed to decline. The rate of this decline differs greatly between people, with genes and lifestyle playing important roles. One gene linked to Alzheimer’s disease risk is called APOE. People who carry the ε4 version of this gene are at a greater risk of cognitive decline. However, carrying the ε2 version may offer some protection. These genetic effects can also differ between females and males. In this study, about 3,000 Black and White adults were followed over 10 years to understand how APOE gene versions, biological sex, and physical activity interact to influence thinking abilities in their 70 and 80s. Participants also reported the number of minutes spent walking per week across the study on average. We found that both females and males who carried the APOE ε4 version experienced faster declines in thinking abilities. However, for these individuals at the greatest genetic risk, those who maintained their walking levels over time showed much slower declines in memory and thinking speed. We also found that higher walking levels at the start of the study were linked to better thinking abilities in females who carried the APOE ε2 or ε3 versions. Overall, our findings suggest that regular walking may help slow age-related cognitive decline, particularly in people at higher genetic risk for Alzheimer’s disease, and that these benefits can differ between females and males.

**Supplementary Information:**

The online version contains supplementary material available at 10.1186/s13293-026-00860-6.

## Highlights

Global cognition and executive functions were assessed over 10 years in community dwelling older adults.

APOE ε4 carriers displayed steeper declines in cognition in both sexes compared to APOE ε3.

APOE ε2 was protective for global cognition in females only .

For both sexes, more walking in APOE ε4 carriers was associated with attenuated cognitive decline.

Walking is an accessible, targeted strategy to slow cognitive decline in older adults at elevated genetic risk.

**Supplementary Information:**

The online version contains supplementary material available at 10.1186/s13293-026-00860-6.

## Background

Cognitive functioning typically declines in advanced age, with some domains more adversely affected, including executive functions, memory, and processing speed [1]. Among older adults, cognitive decline can vary widely with genetic and lifestyle factors playing a considerable role in determining the onset, degree, and severity of impairment [2]. A key genetic factor that influences Alzheimer’s disease risk and also cognitive performance in healthy older adults is the apolipoprotein E (*APOE*) gene [3], which exists in three primary allelic forms (i.e., *APOE* ε2, ε3, and ε4) [3]. Possessing an *APOE* ε4 allele is a risk factor for late-onset Alzheimer’s disease [4], particularly in females [5], and accelerated cognitive decline [6] in comparison to having the *APOE* ε3 allele, the most common and neutral variant [3]. While much less studied, *APOE* ε2 has been associated with protection against Alzheimer’s disease and cognitive decline [5, 7–9], as well as retaining larger gray matter volume in AD-related brain regions, including the hippocampus [10].

Emerging evidence indicates that the *APOE* gene exerts a differential impact on cognitive decline and risk of Alzheimer’s disease by sex [11–14]. For instance, one investigation compiling data from four cohort studies identified a greater protective effect on episodic memory composite score in male *APOE* ε2 carriers than in female *APOE* ε2 carriers [11]. In another study, *APOE* ε4 was linked to lower baseline memory performance, with stronger effects in females than males, but no sex difference between *APOE* ε2 carriers was found [12]. A study using the United Kingdom Biobank data examined cognitive performance across multiple domains, found *APOE* ε4 was associated with poorer cognitive abilities, but observed no interaction with sex [14]. Finally, in a study examining the role of *APOE* ε4 status in both early- and late-onset Alzheimer’s disease, a significant interaction between sex and *APOE* ε4 status was identified, with female carriers experiencing more pronounced cognitive decline in early- but not late-onset Alzheimer’s disease [13]. Collectively, these results suggest a possible interaction between sex and *APOE* genotype on cognitive performance, cognitive decline and Alzheimer’s disease; however, the specific associations have been inconsistent, potentially due to unaccounted for lifestyle factors.

A strong body of evidence from cohort studies, randomized controlled trials, and meta-analyses supports the benefits of physical activity and exercise for delaying cognitive decline and improving brain health in older age [15, 16], with some suggestion of greater benefits in females [15, 17]. However, these studies have largely explored the effect of physical activity on cognitive health at the population level, often overlooking the potential moderating role of genetics [18]. Although *APOE* ε4 has been linked to accelerated cognitive aging [5, 7–9], the evidence on whether exercise can offset its negative effect remains mixed [19, 20]. Some observational studies indicate regular physical activity may mitigate the cognitive detriments associated with *APOE* ε4 [19, 20]; however, other studies have failed to replicate this association [21–25]. For example, a six-month aerobic training randomized controlled trial found smaller improvements in executive functions among *APOE* ε4 carriers compared to non-carriers [26], and a larger multidomain lifestyle intervention that included exercise reported no differences in efficacy based on *APOE* status [27]. Conceivably, these inconsistent findings may reflect sex differences in the relationship between *APOE* ε4 and efficacy of physical activity and exercise for protecting against cognitive decline. One investigation noted that while lifestyle activities generally supported cognitive reserve, females with *APOE* ε4 carrier status experienced an attenuated benefit [28]. Given that *APOE* ε4 accounts for nearly 50% of the genetic risk for late-onset Alzheimer’s disease with greater effects in females [5], clarifying how physical activity interacts with both APOE status and sex is critical. This nuanced understanding could pave the way for better tailored interventions, optimizing exercise prescriptions to bolster cognitive health across different populations.

Thus, this study aims to: (1) investigate the relationship between *APOE* genotype, biological sex, and cognitive performance over a 10-year time span; and (2) examine if habitual physical activity in the form of walking moderates these relationships in initially well-functioning black and white older adults.

## Methods

### Study design and participants

Health ABC is a prospective cohort study of 3,075 community-dwelling black and white older individuals without dementia at baseline from Memphis, TN or Pittsburgh, PA aged 70–79 years at study entry. Cognitive tracking began in 1997 in eligible participants. The primary analyses involved 2,985 of the 3,075 participants with baseline genotyping data, with 90 participants not included as they possessed both *APOE* ε2 and ε4 alleles. However, subsequent exploratory analyses were completed using all six APOE genotypes. The University of Tennessee Memphis, the University of Pittsburgh, and the University of California San Francisco gave study approval, and participants completed written informed consent prior to study commencement.

### Descriptive variables and covariates

Demographic and health characteristics were collected in 1997/1998 (i.e., baseline). Demographics included biological sex (female/male), age, race (black/white), educational attainment (did not complete high school, completed high school, education beyond high school), body mass index (BMI; kg/m^2^), and study site (Memphis/Pittsburgh). Biological sex (female/male) was used in accordance with CIHR sex- and gender-based analysis reporting guidelines. Health characteristics included the presence of coronary heart disease, congestive heart failure, diabetes, cerebrovascular disease, peripheral vascular disease, and pulmonary disease, which were determined at baseline through a combination of self-reported medical history, physician-diagnosed conditions, and medication use. These conditions were combined into a composite health status variable, with scores ranging from 0 to 6, with a higher scores indicating greater comorbidity.

### Cognitive functioning

The Digit Symbol Substitution Test (DSST) was used to assess sustained attention, working memory and information processing, at baseline (year 1) and subsequently at years 5, 8, and 10 [29–32]. Participants were given 90 s to complete as many digit-symbol pairings as possible from a set of 9 pairs, with the total number of correctly coded items constituting the DSST score. Higher scores reflect better functioning. Global cognitive performance was evaluated using the Modified Mini-Mental Status Examination (3MS) [33] in years 1, 3, 5, 8, and 10. The 3MS score ranges from 0 to 100, with scores below 80 indicating cognitive impairment. For the DSST and 3MS, linear mixed effect models (lme4 package; R version 3.4.3) were used to estimate individual linear slopes of performance over time (change in performance over time) and their intercept (estimated initial performance at baseline). These initial values and slopes were calculated using the entirety of the Health ABC cohort to maximize the robustness of these calculations. This approach enabled inclusion of participants with data missing at random (e.g., a skipped visit).

### DNA collection and APOE genotyping

The *APOE* genotype was identified through genetic analysis of blood samples in line with published recommendations [34, 35] and previous Health ABC papers [36, 37]. DNA was extracted from whole-blood samples using Gentra Systems (Minneapolis, MN). Two single nucleotide polymorphisms (SNPs), rs429358 and rs7412, were used to distinguish the ε2, ε3, and ε4 alleles [36, 37]. The genotyping process involved polymerase chain reaction (Pcr) amplification followed by restriction enzyme digestion. This method enabled the classification of the major *APOE* isoforms based on these key genetic variants. The *APOE* genotypes were categorized into six possible combinations based on the alleles: e2/ε2, ε2/ε3, ε2/ε4, ε3/ε3, ε3/ε4, and ε4/ε4. These were collapsed into three groups: *APOE* ε2 (ε2/ε2 and ε2/ε3) *APOE* ε3 (ε3/ε3), and *APOE* ε4 (ε3/ε4 and ε4/ε4), while those with the ε2/ε4 alleles were excluded (*n* = 90). The distribution of the six *APOE* genotypes did not deviate from Hardy-Weinberg Equilibrium, as confirmed by a chi-square goodness-of-fit test with 3 degrees of freedom (^2^ = 1.15, = 0.765) [38].

### Physical activity

Self-reported walking time (minutes per week) was measured annually from year 1 to year 10 using a standardized questionnaire specifically designed for Health ABC based on a previously established questionnaire [39]. Similar to cognition, both initial walking time (i.e., initial values at baseline) and change in walking over time (i.e., slope) were computed using linear mixed model calculations as previously done [40].

### Statistical analysis

Statistical analyses were computed using RStudio (version 4.4.2). The mice package was used for missing data imputation [41]. Chi-square tests were completed using the rstatix package [42]. Linear models were conducted with the base lm function, with the emmeans package [43] used to obtain estimated marginal means. Latent growth curve modeling was carried out with the lavaan package [44].

Missing data were handled using the Multivariate Imputation by Chained Equations (MICE) method, which generates multiple imputations for each missing value based on predictive models constructed from the observed data (< 6% missing data across all variables). For this study, the imputation model included all key variables of interest, including *APOE* genotype, cognitive performance metrics [intercept (estimated initial performance at baseline) and slope values], walking parameters [intercept (initial) and slope], and covariates such as age, sex, education, BMI, study site, and the composite health score components. Predictive mean matching was chosen as the imputation method due to its robustness for handling semi-continuous and continuous variables. Twenty-five imputed datasets were generated and analyzed separately, with pooled results obtained using Rubin’s rules to ensure robust and unbiased estimates.

Chi-square tests or unpaired tests with Cohen’s *d* effect sizes were conducted to examine differences in demographic variables between sexes for categorical and numeric data, respectively. To evaluate the relationship between *APOE* genotype, sex, and cognitive performance [intercepts (initial) and slope values], linear models were conducted. These models included *APOE* genotype as the primary predictor, with sex-specific *APOE* terms (dummy coded) to assess differences within each sex (reference: female *APOE* ε3 and male *APOE* ε3) with adjustment for race (reference: black), study site (reference: Memphis), age, BMI, education (reference: completed high school), and composite health score. Moreover, from these models, estimated marginal means and 95% confidence intervals were calculated for each sex and *APOE* genotype to facilitate comparisons between sex and genotype over 10 years of follow-up. These sex-stratified analyses were subsequently repeated using all six APOE genotypes with ε3/ε3 as the reference group (Supplemental Table 1).

To assess the moderating role of habitual physical activity in the form of self-reported time spent walking on the relationship between *APOE* genotype and cognition, latent growth curve models were employed. Separate models were constructed to examine initial habitual physical activity (initial walking time) on initial cognition and the change in habitual physical activity (slope of walking time) on the change in cognition over the 10-year follow-up. Walking terms were included as interaction terms with *APOE* groups, allowing group-specific effects to be estimated. Each *APOE* genotype group (*APOE* ε2, *APOE* ε3, *APOE* ε4) was represented as a dummy variable and included as an interaction with the walking term. Latent growth curve models were stratified by sex, with adjustments for race, study site, composite health score, age, education, and BMI.

Finally, to compare between sexes, z-scores based on the estimated marginal means and standard errors for each APOE genotype group were computed within males and females from both the main linear regressions and the latent growth curve models. These z-tests quantified the statistical significance of sex differences in cognitive scores associated with each APOE genotype (Supplemental Table 2). This process was repeated for race, where the linear and latent growth curve models were sex- and race-stratified, and subsequently compared using z-tests. This is presented and discussed in the supplemental material (Supplemental Table 3). Alpha was set a priori at 0.05.

## Results

### Demographics

Participant demographics and characteristics are shown in Table 1. A sex difference was noted for the distribution of black and white participants between sexes (χ²=25.7, *p* < 0.001) with males having a higher proportion of white participants than females. Females were older than males (t = 3.25, *p* < 0.001, Cohen’s *d* = 0.13) and had a lower body mass index (t = 3.17, *p* = 0.002, Cohen’s *d* = 0.12) albeit these had negligible effect sizes. For study site, no difference was observed by sex (χ²=0.05, *p* = 0.822). Educational attainment differed between sexes (χ²=55.7, *p* < 0.001), with males and black participants more likely to have not completed high school. *APOE* genotype also showed significant differences by sex (χ²=8.51, *p* = 0.014), with males having a higher prevalence of the *APOE* ε2, white participants having a higher prevalence of *APOE* ε3, and black participants having a higher prevalence of *APOE* ε4. Composite health scores differed between sexes (χ²=18.3, *p* = 0.002) with females and white participants having lower composite health scores (i.e., fewer health comorbidities). Finally, females had lower initial habitual walking (t = 9.39, *p* < 0.001, Cohen’s *d* = 0.34) and less decline over time (t = 1.93, *p* = 0.054, Cohen’s *d* = 0.07) than males, though effect sizes were small and negligible.

**Table 1:** Demographics and clinical characteristics by sex

Variable	Females (*n* = 1,537)	Males (*n* = 1,444)	Sex Comparison
Race			χ2 = 25.7, *p* < 0.001
Black	692 (45.0%)	519 (35.8%)	
White	845 (55.0%)	929 (64.2%)	
Age	73.8 (2.9)	73.5 (2.9)	t = 3.25, *p* < 0.001, *d* = 0.13
Body Mass Index	27.1 (4.0)	27.6 (5.5)	t = 3.17, *p* = 0.002, *d* = 0.12
Site			χ^2^ = 0.02, *p* = 0.892
Memphis	770 (50.1%)	730 (50.4%)	
Pittsburgh	767 (49.9%)	718 (49.6%)	
Education Attained			χ^2^ = 57.1, *p* < 0.001
Not Completed High School	381 (26.3%)	600 (39.0%)	
Completed High School	382 (26.4%)	367 (23.9%)	
Greater than High School	685 (47.3%)	570 (37.1%)	
*APOE* Grouping			χ^2^ = 6.37, *p* = 0.041
* APOE* ε2	233 (16.1%)	205 (13.3%)	
* APOE* ε3	846 (58.4%)	894 (58.2%)	
* APOE* ε4	369 (25.5%)	438 (28.5%)	
Health Characteristics			
Coronary Heart Disease	338 (23.3%)	170 (11.1%)	
Congestive Heart Failure	29 (2.0%)	13 (0.8%)	
Diabetes	255 (17.6%)	205 (13.3%)	
Pulmonary Disease	387 (26.7%)	233 (15.2%)	
Cerebrovascular Disease	99 (6.8%)	116 (7.5%)	
Peripheral Vascular Disease	92 (6.4%)	58 (3.8%)	
Composite Health Score			χ^2^ = 103.7, *p* < 0.001
Zero	637 (44.0%)	924 (60.1%)	
One	528 (36.5%)	463 (30.1%)	
Two	196 (13.5%)	121 (7.9%)	
Three or More	87 (5.9%)	29 (2.0%)	
Walking Minutes			
Year 1	111 (281)	168 (388)	
Year 2	105 (214)	154 (284)	
Year 3	72 (134)	123 (198)	
Year 4	70 (169)	111 (267)	
Year 5	67 (142)	103 (172)	
Year 6	53 (130)	96 (222)	
Year 7	54 (137)	82 (145)	
Year 8	55 (112)	85 (157)	
Year 9	64 (165)	92 (222)	
Year 10	54 (107)	82 (150)	

Data are displayed as number (percentage) or mean (standard deviation) where appropriate. The health composite is the total number of underlying health factors listed under health characteristics

### Sex-specific relationships between APOE genotype and cognition

#### Digit symbol substitution test

**Fig. 1:**
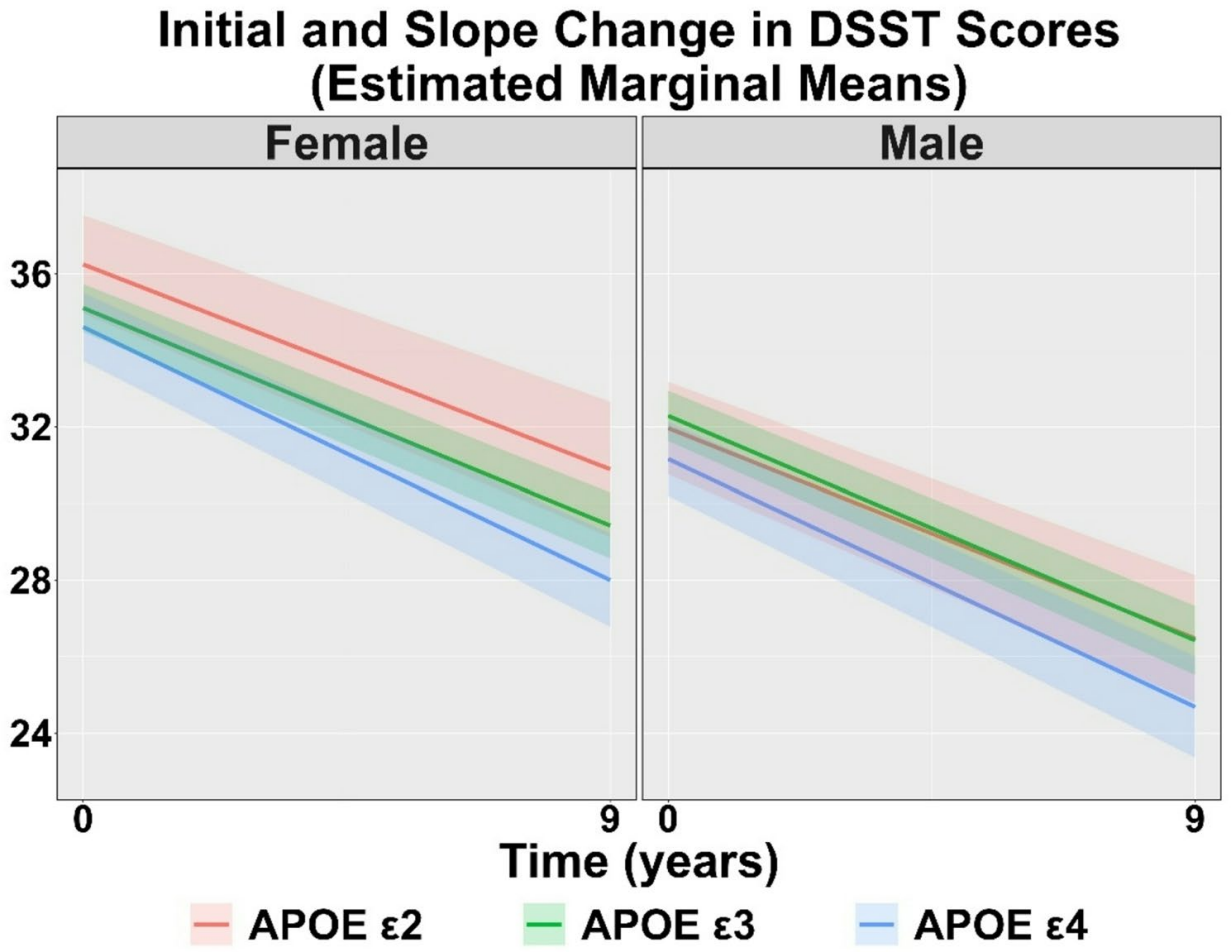
Estimated marginal means and 95% confidence intervals (shaded areas) for Digit Symbol Substitution Test (DSST) cognitive trajectories over 10 years for *APOE e2*,* e3*, and *e4* genotypes in females and males, after adjusting for race, study site, age, education, and the health composite score. In both females and males, *APOE e2* carriers and *APOE e3* carriers did not differ at baseline (initial) or in rate of decline over 10 years. In males only, *APOE e4* carriers showed lower baseline performance than *APOE e3* carriers. In both females and males, *APOE e4* carriers had faster rates of decline over 10 years compared with *APOE e3* carriers

Table 2 displays the raw DSST scores over the course of the study by sex and APOE group. Figure 1 illustrates the estimated marginal mean initial score and change over time (slope) for DSST by *APOE* genotypes for females and males, while Table 3 displays the linear regression outputs. Compared to *APOE* ε3 females, DSST initial scores did not differ for *APOE* ε2 females (β = 1.14, *p* = 0.117) or *APOE* ε4 females (β=-0.50, *p* = 0.366). Compared to *APOE* ε3 males, *APOE* ε4 males had lower DSST initial scores (β=-1.13, *p* = 0.045), while *APOE* ε2 males did not differ (β=-0.32, *p* = 0.645). For DSST slopes, compared to *APOE* ε3 females, *APOE* ε4 females demonstrated a steeper decline (β=-0.10, *p* < 0.001), while *APOE* ε2 females did not differ (β = 0.04, *p* = 0.230). Similarly, compared to *APOE* ε3 males, *APOE* ε4 males demonstrated steeper declines (β=-0.07, *p* = 0.005), while *APOE* ε2 males did not differ (β = 0.04, *p* = 0.152).

**Table 2:** Mean and standard deviation for the Digit Symbol Substitution Test (DSST) and Modified Mini-Mental Status Examination (3MS) across time points across sexes and APOE genotypes

Test	Sex	Year	APOE ε2	APOE ε3	APOE ε4
DSST	Female	1	37.1 (14.4)	37.7 (14.3)	35.5 (15.6)
		5	35.5 (13.5)	35.0 (13.9)	31.9 (14.8)
		8	33.3 (13.6)	33.2 (14.2)	29.7 (14.5)
		10	32.5 (13.6)	32.2 (13.8)	28.3 (14.6)
	Male	1	31.8 (15.1)	35.1 (14.0)	32.0 (15.0)
		5	30.0 (14.5)	32.8 (13.9)	29.0 (14.8)
		8	28.5 (14.4)	30.4 (13.6)	26.2 (14.5)
		10	27.1 (13.6)	29.4 (13.4)	25.4 (14.1)
3MS	Female	1	90.7 (7.7)	91.2 (7.3)	89.2 (9.8)
		3	90.2 (8.2)	90.5 (7.9)	87.5 (10.9)
		5	90.0 (8.8)	90.0 (8.6)	86.9 (12.0)
		8	88.5 (9.2)	87.6 (10.1)	84.3 (12.8)
		10	87.9 (10.2)	86.8 (11.0)	83.0 (14.0)
	Male	1	89.2 (9.6)	90.3 (7.9)	87.6 (9.5)
		3	88.6 (9.8)	89.8 (8.6)	86.1 (11.1)
		5	88.7 (9.9)	90.2 (8.5)	86.6 (10.5)
		8	86.4 (11.4)	87.3 (9.8)	82.4 (13.3)
		10	86.1 (11.3)	87.0 (10.3)	81.4 (13.9)

**Table 3:** Full outputs for the linear models stratified by sex examining APOE genotype differences in cognitive outcomes

	DSST Initial	DSST Slope	3MS Initial	3MS Slope
Female				
* APOE* ε3 (Reference)				
* APOE* ε2	β = 1.14; *p* = 0.117	β = 0.04; *p* = 0.230	β = 0.38; p = 0.381	β = 0.15; *p* = 0.002
* APOE* ε4	β=-0.50; *p* = 0.366	**β=-0.10; p = < 0.001**	**β=-0.98; p = 0.003**	**β=-0.13; p = < 0.001**
Male				
* APOE* ε3 (Reference)				
* APOE* ε2	β=-0.32; p = 0.645	β = 0.04; *p* = 0.152	β = 0.21; *p* = 0.615	β = 0.06; *p* = 0.215
* APOE* ε4	**β=-1.13; p = 0.045**	**β=-0.07; p = 0.005**	**β=-1.66; p = < 0.001**	**β=-0.22; p = < 0.001**
Race (Reference: Black)	**β = 9.03; p = < 0.001**	β=-0.00; p = 0.776	**β = 4.54; p = < 0.001**	**β = 0.22; p = < 0.001**
Site (Reference: Memphis)	**β = 3.38; p = < 0.001**	β = 0.02; *p* = 0.105	**β = 1.43; p = < 0.001**	**β = 0.29; p = < 0.001**
Age	**β=-0.83; p = < 0.001**	**β=-0.01; p = < 0.001**	**β=-0.32; p = < 0.001**	**β=-0.04; p = < 0.001**
Education				
Completed High School (Reference)				
Did not Complete High School	**β=-7.68; p = < 0.001**	β = 0.01; *p* = 0.543	**β=-4.20; p = < 0.001**	**β=-0.16; p = < 0.001**
Greater than High School	**β = 4.46; p = < 0.001**	β = 0.02; *p* = 0.176	**β = 2.56; p = < 0.001**	**β = 0.14; p = < 0.001**
Health Score Composite	**β=-1.55; p = < 0.001**	**β=-0.02; p = 0.007**	**β=-0.35; p = 0.005**	**β=-0.03; p = 0.011**

Significant associations are presented in boldface. Digit Symbol Substitution Test (DSST), Modified Mini-Mental Status Examination (3MS), and Body Mass Index (BMI)

#### Modified mini-mental status examination

**Fig. 2:**
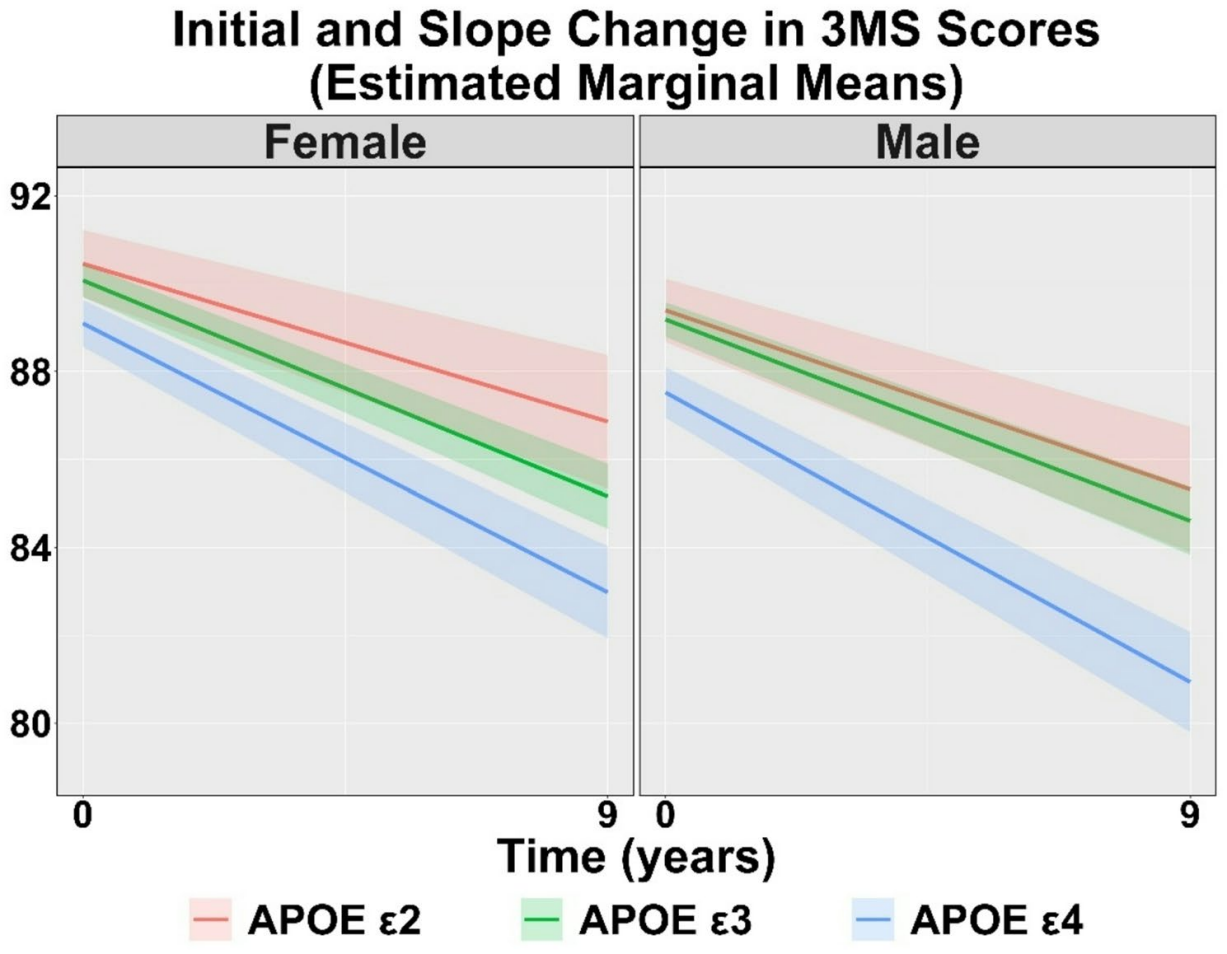
Estimated marginal means and 95% confidence intervals (shaded areas) for the Modified Mini-Mental State Examination **(**3MS) cognitive trajectories over 10 years for *APOE e2*,* e3*,* and e4* genotypes in females and males, after adjusting for race, site, age, education, and the health composite score. In both females and males, *APOE e4* carriers showed lower baseline (initial) performance and faster rates of decline over 10 years compared with *APOE e3* carriers. In females only, *APOE e2* carries showed slower rates of decline than *APOE e3* carriers

Table 2 displays the raw 3MS scores over the course of the study by sex and APOE group. Figure 2 shows the estimated marginal mean initial score and change over time (slope) for 3MS by *APOE* genotypes for females and males, while Table 3 displays the linear regression outputs. Compared to *APOE* ε3 females, *APOE* ε4 females had lower initial 3MS scores (β=-0.98, *p* = 0.003), while *APOE* ε2 females did not differ (β = 0.38, *p* = 0.381). Similarly, compared to *APOE* ε3 males, *APOE* ε4 males (β=-1.66, *p* < 0.001) had lower 3MS initial scores, while *APOE* ε2 males did not differ (β = 0.21, *p* = 0.615). For 3MS slopes, compared to *APOE* ε3 females, *APOE* ε4 females (β=-0.13, *p* < 0.001) demonstrated steeper declines, while *APOE* ε2 females had a slower rate of decline (β = 0.15, *p* = 0.002). Compared to *APOE* ε3 males, *APOE* ε4 males showed steeper declines in 3MS (β=-0.22, *p* < 0.001), while *APOE* ε2 males did not differ (β = 0.06, *p* = 0.215).

### Protective role of habitual physical activity on cognition is APOE genotype- and sex-specific

#### Initial time spent walking and initial cognitive score

**Fig. 3:**
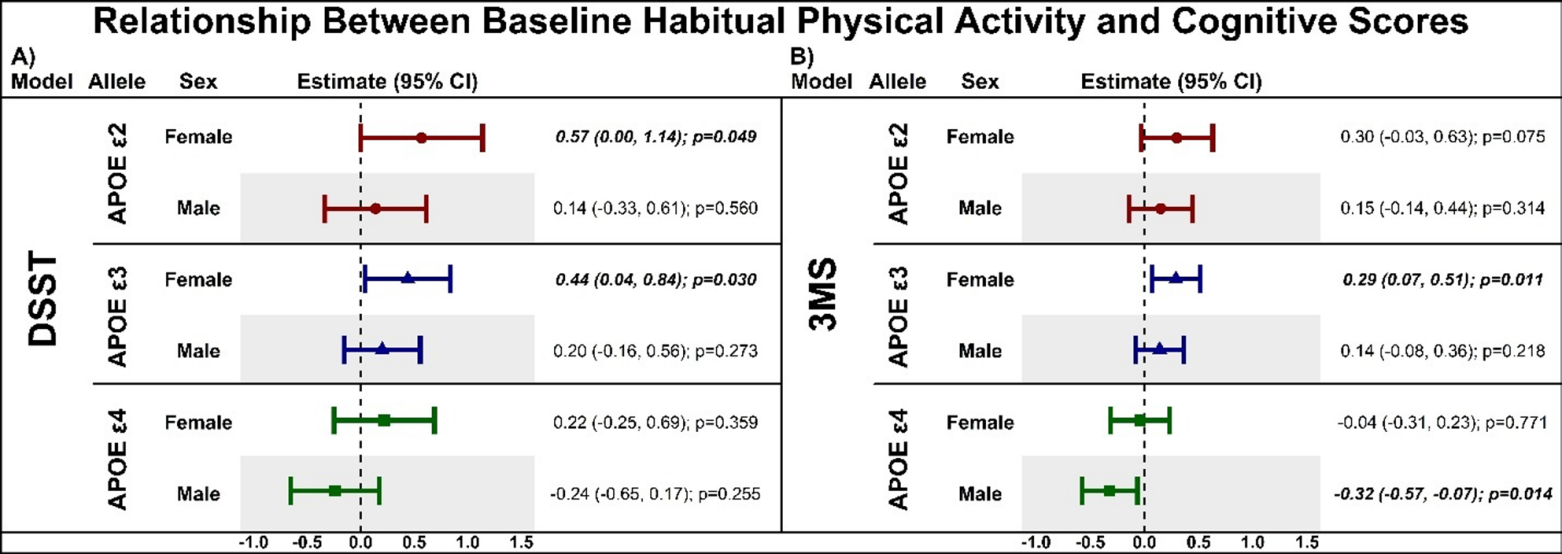
Latent growth curve estimates displaying the association between baseline (initial) habitual physical activity in the form of self-reported time spent walking and baseline (initial) cognitive performance on the Digit Symbol Substitution Test (panel A) and the Modified Mini-Mental State Examination (panel B) as a function of *APOE* genotype, stratified by sex. Data are displayed as estimates with 95% confidence intervals (CI) with significant values bolded and italicized. **A**). Only *APOE e2* and APOE *e3* female carriers showed an significant association between greater initial time spent walking and initial DSST performance. **B**). Greater initial time spent walking was associated with higher initial 3MS performance in female *APOE e3* and *APOE e2* carriers and lower initial 3MS performance in male *APOE e4* carriers. Digit Symbol Substitution Test (DSST), Modified Mini-Mental Status Examination (3MS)

Figure 3A shows the relationship between initial time spent walking and initial score on the DSST as a function of *APOE* genotype in females and males. Greater initial time spent walking was positively associated with higher initial DSST score for *APOE* ε2 females (β = 0.57, *p* = 0.049) and *APOE* ε3 females (β = 0.44, *p* = 0.030), with no relationship seen in *APOE* ε4 females (*p* = 0.359). In males, greater initial time spent walking was not associated with initial DSST score regardless of APOE genotype (all *p* > 0.254).

Figure 3B shows the relationship between initial time spent walking and initial 3MS score as a function of APOE genotype in females and males. Greater initial time spent walking was associated with higher initial 3MS score for *APOE* ε3 females (β = 0.29, *p* = 0.011) and a trend in *APOE* ε2 females (β = 0.30, *p* = 0.075), with no relationship was observed in *APOE* ε4 females (*p* = 0.771). In males, greater initial time spent walking was associated with lower initial 3MS score for *APOE* ε4 males (β=-0.32, *p* = 0.014), with no relationships seen in *APOE* ε2 and ε3 carriers (both p’s > 0.217)

#### Change in time spent walking and change in cognitive scores over 10 years

**Fig. 4:**
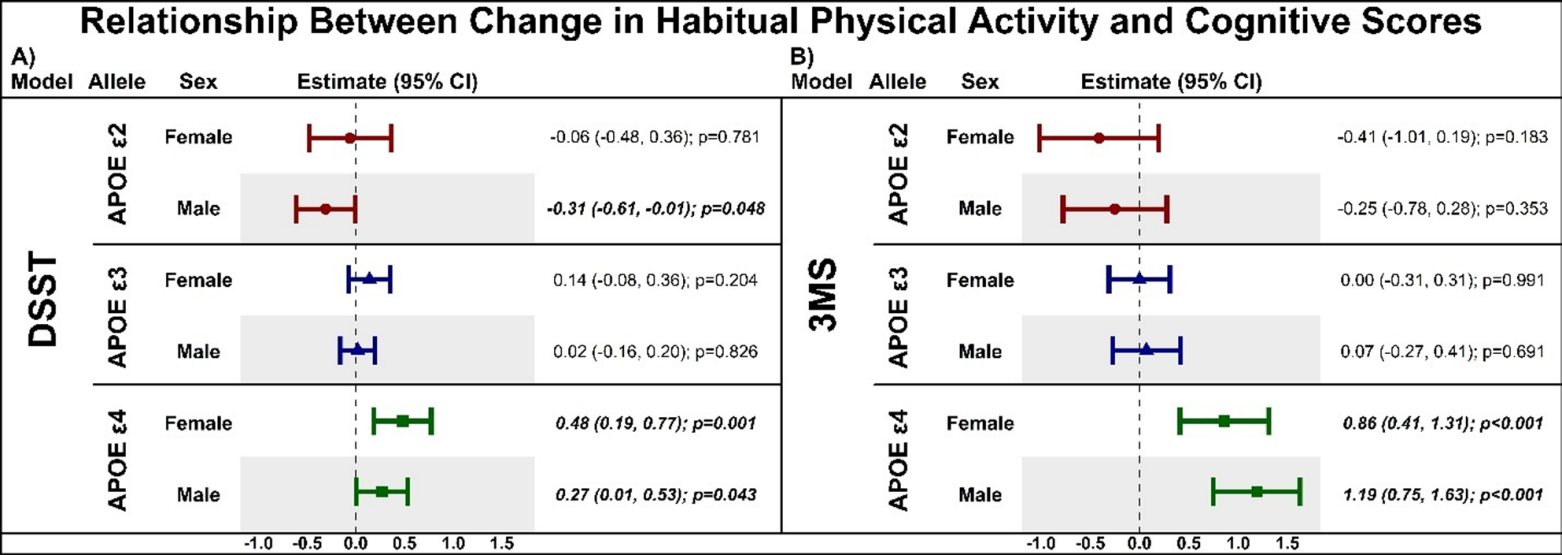
Latent growth curve estimates displaying the association between change in habitual physical activity in the form of self-reported time spent walking over 10 years and change in cognitive performance on the Digit Symbol Substitution Test (panel A) and the Modified Mini-Mental State Examination (panel B) as a function of *APOE* genotype, stratified by sex. Data are displayed as estimates with 95% confidence intervals (CI) with significant values bolded and italicized. (**A**) Increases or maintenance of time spent walking was associated with less decline in DSST performance in both male and female *APOE e4* carriers and greater declines in *APOE e2* males. (**B**) Increases or maintenance of time spent walking was associated with less decline in 3MS performance in both male and female *APOE e4* carriers. Digit Symbol Substitution Test (DSST), Modified Mini-Mental Status Examination (3MS)

Figure 4A shows the relationship between change in time spent walking and change in DSST over 10 years as a function of *APOE* genotype in females and males. Greater increases or maintenance of walking over time was associated with less decline in DSST performance in *APOE* ε4 carriers for both females (β = 0.48, *p* = 0.001) and males (β = 0.27, *p* = 0.043). In *APOE* ε3 female and male carriers, no associations were found between change in walking and change in DSST (both p’s > 0.203). In *APOE* ε2 males only, greater increases or maintenance of walking was associated with greater declines in DSST (β=-0.31, *p* = 0.048), with no association in females (β = 0.06, *p* = 0.781).

Figure 4B shows the relationship between change in time spent walking and change in 3MS over 10 years as a function of *APOE* genotype in females and males. Similar to the results seen for DSST change in performance, greater increases or maintenance of walking was associated with less decline in 3MS performance in *APOE* ε4 carriers for both females (β = 0.86, *p* < 0.001) and males (β = 1.19, *p* < 0.001). For both female and male *APOE* ε2 and ε3 carriers, no significant associations were found between change in walking and change in 3MS (all p’s > 0.182).

## Discussion

Although the risk of dementia associated with the *APOE* ε4 genotype is well-established, the degree to which habitual physical activity may moderate this genetic risk has not been adequately interrogated. Findings from the current study are encouraging as greater yearly maintenance of weekly walking appears to protect against declines in global cognition and executive functioning in both female and male *APOE* ε4 carriers. Overall study findings reveal somewhat complex interactions between *APOE* genotype, sex, and physical activity for cognition, suggesting that while male and female *APOE* ε4 carriers demonstrate greater cognitive decline, engaging in habitual physical activity appears to mitigate these effects.

### Sex specific cognitive trajectories linked to APOE ε2 protection and APOE ε4 decline

Our finding that possessing an *APOE* ε4 allele has a detrimental effect on cognitive function aligns with previous studies [45, 46]. While we observed this detrimental effect in both sexes, other studies suggest the negative influence of the ε4 allele is greater in females [47]. In a study focused on baseline performance, *APOE* ε4 was linked to poorer memory outcomes, with a stronger effect in females than in males [12]. However, no sex differences were present for executive functions or language scores across all *APOE* genotypes [12]. Our supplementary analysis additionally identified that white participants had higher initial DSST and 3MS scores and less decline in the 3MS slope compared to black participants. Thus, the interaction between sex, race, and *APOE* genotype may be cognitive domain-specific, warranting further investigation.

Although our analysis did not directly compare scores between sexes, in a mixed-race cohort we found a protective effect of *APOE* ε2 only in females. Previous work examining the *APOE* ε2 allele has been mixed, with some reporting no protective effect [12], some reporting a protective effect in females [12, 48] or males [11, 12] only depending on the cognitive domain analyzed. Importantly, a meta-analysis across 27 independent research studies demonstrated that while the APOE ε2/ε3 genotype was protective against cognitive decline for both females and males, the magnitude was greater within females [48]. Thus, while mixed, the available literature suggests the neuroprotective effect of exercise against cognitive decline may be stronger within females.

### Walking is protective against declines in cognition in APOE e4 carriers

We found that greater maintenance of minutes per week of walking protects against declines in both global cognition and executive functioning/processing speed in male and female *APOE* ε4 carriers. Importantly, these protective associations were comparable among Black and White participants, indicating similar benefits across racial groups (Supplemental Table 3). Exploratory calculations identified that in order for these carriers to avoid cognitive decline, they needed to maintain ~ 94% of their prior years walking. This provides further support that *APOE* ε4 carriers are susceptible to neurodegeneration and, therefore, may derive greater benefit from physical lifestyle interventions [49]. *APOE* ε4 is known to exacerbate neurodegenerative processes by promoting greater amyloid-beta accumulation and deposition in the brain, leading to elevated neuroinflammation/oxidative stress [50]. Pre-clinical work has demonstrated that male mice with the human *APOE* ε4 allele have lower vascular density than mice with the human *APOE* ε3 allele [51]. Moreover, in humans greater regional decline in cerebral blood flow has been noted in ε4 carriers relative to non-carriers [52, 53]. Together, these findings observed across different study designs, suggest differential vascular responses are consistently seen in ε4 carriers relative to other *APOE* genotypes. The *APOE* ε4 allele negatively affects blood-brain barrier integrity, which additionally influences cognitive decline by allowing harmful substances to enter the brain parenchyma more readily [54]. This compromised blood-brain barrier integrity, in conjunction with reduced cerebral vascular density and aberrant blood flow patterns, highlights the multifaceted vulnerabilities in *APOE* ε4 carriers that predispose them to accelerated neurodegeneration.

Physical activity works to mitigate these detrimental effects through several mechanisms. For example, physical activity or structured aerobic exercise enhances cerebral blood flow across the lifespan, which improves oxygen and nutrient delivery to brain tissues, as well as increasing circulating levels of brain derived neurotrophic factor (BDNF), a key molecule involved in promoting neurogenesis and synaptic plasticity [55]. Physical activity and aerobic exercise also promote angiogenesis, which ensures adequate perfusion of the brain parenchyma, and reduce neuroinflammation and oxidative stress by promoting anti-inflammatory cytokine profiles and enhancing antioxidant defenses [56–59]. Collectively, these mechanisms work to preserve cognitive function by mitigating the negative consequences of the aging process.

*APOE* ε4 carriers have lower levels of BDNF in circulation than non-carriers in both cognitively normal older adults and those with Alzheimer’s disease [60, 61]. Thus, it could be the case that engaging in greater walking could be more beneficial in *APOE* ε4 carriers through a greater net increase in BDNF levels. While understudied, results from a study with a very small sample of older adults with mild cognitive impairment do not support this, as 6-months of aerobic exercise suggested greater BDNF increases in *APOE* ε4 non-carriers [62]. However, conclusions must be cautiously drawn from this study as only 13 participants were randomized to the aerobic exercise group and 7 were ε4 carriers. Thus, future studies with much larger sample sizes are needed. Physical activity directly addresses this specific vulnerability in *APOE* ε4 carriers, offering a potential target for interventions to counteract the accelerated cognitive decline associated with the *APOE* ε4 genotype [8]. *APOE* e4 has been shown to increase amyloid beta aggregation, tau pathology, and neuroinflammation [60]; engagement in higher levels of physical activity for humans and rodents can decrease all three [63–65]. However, our understanding of why exercise-induced reductions in amyloid beta, tau pathology, and neuroinflammation may differ by biological sex remains limited, emphasizing a critical gap in current research. These findings also suggest a potential genotype-specific dose-response relationship exists, where *APOE* ε4 carriers may derive cognitive benefit from lower intensities or volumes of physical activity. Conversely, *APOE* ε2 and *APOE* ε3 carriers may require higher activity thresholds to achieve comparable cognitive benefits. This may, in part, explain the negative association between greater walking and DSST performance observed in male *APOE* ε2 carriers (Fig. 4), indicating that walking alone may be insufficient to confer benefits to sustained attention and processing speed, with greater activity intensity or additional lifestyle factors likely required for this group. Hence, future research using objective measures and/or validated questionnaires that capture physical activity volume, domain, and frequency may facilitate the development of genotype-specific physical activity guidelines to support long-term brain health.

### Greater walking at baseline associated with better baseline cognition in a sex- and APOE genotype-specific manner

We found greater initial walking was associated with higher initial global cognition and executive functions scores for *APOE* ε2 and ε3 carriers but not ε4 carriers. This was seen only in females. The majority of previous work examined the relationship between physical activity and cognition only in ε4 carriers vs. non-carriers [19–25]. To our knowledge, no other analyses have examined the relationship between physical activity and cognition in *APOE* ε2 carriers, with or without consideration of biological sex. Interestingly, in older aged males who were physically active at baseline, *APOE* ε2 and ε3 carriers were at lower risk of mortality after 18-years, whereas ε4 carriers were at increased risk; similar relationships were not observed in older females [66].

APOE genotypes differentially influence cognitive aging partly through their role in lipid metabolism. Apolipoprotein E protein (ApoE) isoforms differ in lipidation [8], with ApoE4 being poorly lipidated and more likely to bind low-density lipoprotein (LDL) cholesterol, while ApoE2 and ApoE3 isoforms preferentially binding high-density lipoprotein (HDL) cholesterol [67]. This contributes to higher LDL and lower HDL levels in *APOE* e4 carriers, which are patterns linked to cognitive decline, particularly in postmenopausal females [68–70]. Physical exercise is known to improve lipid profiles, which may be especially beneficial for cognitive health in *APOE* ε4 carriers [71, 72] possibly in a sex-specific manner [73]. Future research should explore whether exercise can offset genotype- and sex-related lipid differences to support brain health.

### Limitations and future directions

A limitation of the current study is reliance on self-reported walking data to assess the role of physical activity, which can be susceptible to recall bias [74]. Additionally, activity exposure data has been heterogeneously used across the literature with some studies treating activity as a continuous measure and others attending to a variety of defined categories. Inconsistency in how physical activity is assessed and analyzed across studies hampers meaningful comparisons across studies and could obscure the true relationship between physical activity and cognitive outcomes. Furthermore, walking was examined as a singular form of physical activity, potentially underestimating the broader benefits that might be observed with more comprehensive assessments of moderate-to-vigorous physical activity. Despite these limitations, this study identified a significant protective role of walking in *APOE* ε4 carriers, suggesting that the observed effects may represent a conservative estimate. Had objective measures of moderate-to-vigorous physical activity been collected and included as a moderator of interest, the impact of physical activity on slowing cognitive decline in this high-risk group may have been more pronounced. It is also worth considering that the types of physical activity typically undertaken by males and females may differ in intensity, duration and physiological impact [75], potentially contributing to sex-specific associations with cognitive outcomes.

## Conclusions

This study highlights the complex relationship between *APOE* genotype and biological sex in shaping cognitive trajectories over a 10-year period and the beneficial role of walking. We found male and female *APOE* ε4 carriers exhibited steeper declines in global cognition and executive/processing speed, whereas *APOE* ε2 conferred modest protection for decline in global cognition over the decade of follow-up in females only. Despite the negative trajectory for *APOE* ε4 carriers, physical activity engagement displayed the greatest protective effect for both females and males in this high-risk group. Moreover, higher initial walking levels were associated with better global cognition and executive functions/processing speed for *APOE* ε2 and APOE ε3 females. These results underscore the potential of regular physical activity, as a targeted and accessible intervention, to slow cognitive decline, particularly for those genetically at risk of Alzheimer’s disease.

## Supplementary Information

Below is the link to the electronic supplementary material.

Supplementary Material 1

Supplementary Material 2

Supplementary Material 3

Supplementary Material 4

## Data Availability

The data used in this study were accessed through a restricted-use agreement with the Health ABC Study and thus the authors are not permitted to share these data. Interested researchers may apply for access directly from the Health ABC Study.
