# Supplementary material for: Walking to protect against cognitive decline: the role of APOE genotype and sex
Source: Biol Sex Differ. 2026 Feb 21;17:58. doi: 10.1186/s13293-026-00860-6 (PMC13032221; doi:10.1186/s13293-026-00860-6)
Supplement: Supplementary file 1 — Supplementary Material 1 [file 13293_2026_860_MOESM1_ESM.docx]

**Supplemental Material**

*Results*

*Linear Regression Outputs Stratified Into APOE Diplotypes*

Supplemental Table 1 displays linear regressions cognition outputs comparing APOE diplotypes to the *APOE* ε3/ε3 reference group; however, samples sizes for the ε2/ε2, ε2/ε4, and ε4/ε4 groups were smaller, which may have limited statistical power for these estimates. Compared to *APOE* ε3/ε3 females, ε2/ε3 females had a higher Digit Symbol Substitution Test (DSST) initial score (*p=0.046*); however, this was not different for all other female diplotypes (all *p>0.095*). No difference between diplotypes were found for males (*p>0.102*). For DSST slopes, ε3/ε4 females (*p<0.001*) and male ε2/ε4 (*p=0.048*), ε3/ε4 (*p=0.019*), and ε4/ε4 (*p=0.014*) carriers had a steeper decline relative to their sex-specific reference group. No other diplotypes were different compared to the ε3/ε3 group (all *p>0.053*). For the Modified Mini-Mental Status Examination (3MS) initial scores, male and female ε3/ε4 and ε4/ε4 carriers had lower scores relative to the ε3/ε3 reference group (all *p<0.021*). No differences were present for the ε2/ε2, ε2/ε3, and ε2/ε4 groups (all *p>0.147*). For the 3MS slope, female ε2/ε3 carriers had less decline compared to ε3/ε3 females (p-0.004), while both male and female ε3/ε4 and ε4/ε4 had a steeper decline (all *p<0.015*). No difference was present for the other diplotypes (all *p>0.183*).

*Sex Differences in APOE Genotype Associations with Cognitive Outcomes*

Supplemental Table 2 displays the z-score comparison between sexes from the main sex-stratified linear regression and the walking latent growth curve models. Across genotypes, females exhibited higher initial DSST (all *p<0.001*) and 3MS (all *p<0.048*) scores compared to males; however, both sexes displayed a similar rate of decline across the study for both DSST (all *p>0.292*) and 3MS (all *p>0.219*). The influence of walking was similar between sexes for all cognitive outcomes (all *p>0.136*).

*Race Differences in APOE Genotype Associations with Cognitive Outcomes*

Supplemental Table 3 displays the z-score comparison between black and white participants from the sex- and race-stratified linear regression and walking latent growth curve models. Across genotypes and sexes, white participants had higher initial DSST (all *p<0.001*) and 3MS (all *p<0.001*) and a less steep decline in 3MS scores (all *p<0.002*) compared to the black participants. No difference was present between races in the DSST slope (all *p>0.293*). Additionally, the influence of walking on cognitive outcomes were similar for both races across all cognitive scores, sexes, and genotypes (all *p>0.084*).

|  | **DSST Initial** | **DSST Slope** | **3MS Initial** | **3MS Slope** |
| --- | --- | --- | --- | --- |
| Female |  |  |  |  |
| *APOE* ε3ε3 (Reference) (n=894) |  |  |  |  |
| *APOE* ε2ε2 (n=13) | β=-4.12; p=0.114 | β=0.21; p=0.053 | β=-2.26; p=0.147 | β=0.23; p=0.183 |
| *APOE* ε2ε3 (n=192) | **β=1.49; p=0.046** | β=0.02; p=0.425 | β=0.55; p=0.215 | **β=0.14; p=0.004** |
| *APOE* ε2ε4 (n=47) | β=0.49; p=0.729 | β=-0.06; p=0.282 | β=0.92; p=0.272 | β=-0.04; p=0.643 |
| *APOE* ε3ε4 (n=401) | β=-0.30; p=0.599 | **β=-0.11; p=<0.001** | **β=-0.78; p=0.021** | **β=-0.12; p=0.001** |
| *APOE* ε4ε4 (n=37) | β=-2.62; p=0.095 | β=-0.08; p=0.236 | **β=-3.31; p=<0.001** | **β=-0.32; p=0.002** |
| Male |  |  |  |  |
| *APOE* ε3ε3 (Reference) (n=846) |  |  |  |  |
| *APOE* ε2ε2 (n=14) | β=-1.83; p=0.467 | β=0.15; p=0.140 | β=-0.22; p=0.886 | β=0.09; p=0.563 |
| *APOE* ε2ε3 (n=219) | β=-0.23; p=0.744 | β=0.03; p=0.245 | β=0.22; p=0.605 | β=0.05; p=0.251 |
| *APOE* ε2ε4 (n=43) | β=-2.09; p=0.154 | **β=-0.12; p=0.048** | β=0.87; p=0.319 | β=-0.12; p=0.220 |
| *APOE* ε3ε4 (n=339) | β=-0.98; p=0.102 | **β=-0.06; p=0.019** | **β=-1.53; p=<0.001** | **β=-0.22; p=<0.001** |
| *APOE* ε4ε4 (n=30) | β=-2.82; p=0.103 | **β=-0.18; p=0.014** | **β=-3.37; p=0.001** | **β=-0.28; p=0.015** |
| Race (Reference: Black) | **β=8.98; p=<0.001** | β=-0.00; p=0.756 | **β=4.43; p=<0.001** | **β=0.22; p=<0.001** |
| Site (Reference: Memphis) | **β=3.37; p=<0.001** | β=0.02; p=0.087 | **β=1.40; p=<0.001** | **β=0.29; p=<0.001** |
| Age | **β=-0.82; p=<0.001** | **β=-0.01; p=<0.001** | **β=-0.32; p=<0.001** | **β=-0.04; p=<0.001** |
| Education |  |  |  |  |
| Completed High School (Reference) |  |  |  |  |
| Did not Complete High School | **β=-7.69; p=<0.001** | β=0.01; p=0.625 | **β=-4.23; p=<0.001** | **β=-0.16; p=<0.001** |
| Greater than High School | **β=4.48; p=<0.001** | β=0.02; p=0.178 | **β=2.58; p=<0.001** | **β=0.14; p=<0.001** |
| Health Score Composite | **β=-1.47; p=<0.001** | **β=-0.02; p=0.004** | **β=-0.32; p=0.009** | **β=-0.03; p=0.009** |

**Supplemental Table 1**: Full outputs for the linear models stratified by sex examining APOE genotypes differences in cognitive outcomes.

Digit Symbol Substitution Test (DSST), Modified Mini-Mental Status Examination (3MS), and Body Mass Index (BMI).

**Supplemental Table 2**: Z-score comparisons between males and females from the sex-stratified linear regression and latent growth curve models to quantify sex differences in APOE genotypes associations with cognitive outcomes.

|  | **DSST Initial** | **DSST Slope** | **3MS Initial** | **3MS Slope** |
| --- | --- | --- | --- | --- |
| *Main Regression Analysis* | | | | |
| APOE2 | **z=4.76; p<0.001** | z=0.40; p=0.687 | **z=1.98; p=0.048** | z=0.91; p=0.361 |
| APOE3 | **z=6.11; p<0.001** | z=1.05; p=0.292 | **z=3.23; p=0.001** | z=-1.23; p=0.219 |
| APOE4 | **z=5.16; p<0.001** | z=-0.52; p=0.601 | **z=3.94; p<0.001** | z=1.21; p=0.226 |
| *Walking Latent Growth Curve Modeling* | | | | |
| APOE2 | z=1.12; p=0.264 | z=0.97; p=0.331 | z=0.66; p=0.509 | z=-0.39; p=0.694 |
| APOE3 | z=0.87; p=0.382 | z=0.78; p=0.436 | z=0.92; p=0.355 | z=-0.29; p=0.775 |
| APOE4 | z=1.44; p=0.149 | z=1.04; p=0.298 | z=1.49; p=0.136 | z=-1.03; p=0.303 |

Males served as the reference group. Digit Symbol Substitution Test (DSST) and Modified Mini-Mental Status Examination (3MS).

**Supplemental Table 3**: Z-score comparisons between black and white participants from the sex-stratified linear regression and latent growth curve models to quantify race differences in APOE genotypes associations with cognitive outcomes.

|  | **DSST Initial** | **DSST Slope** | **3MS Initial** | **3MS Slope** |
| --- | --- | --- | --- | --- |
| *Main Regression Analysis* | | | | |
| *Female* | | | | |
| APOE2 | **z=5.16; p<0.001** | z=0.97; p=0.334 | **z=4.47; p<0.001** | **z=3.15; p=0.002** |
| APOE3 | **z=13.92; p<0.001** | z=-0.82; p=0.411 | **z=10.89; p<0.001** | **z=5.56; p<0.001** |
| APOE4 | **z=10.22; p<0.001** | z=0.16; p=0.874 | **z=8.79; p<0.001** | **z=3.58; p<0.001** |
| *Male* | | | | |
| APOE2 | **z=7.23; p<0.001** | z=0.86; p=0.390 | **z=6.80; p<0.001** | **z=3.04; p=0.002** |
| APOE3 | **z=12.87; p<0.001** | z=-0.41; p=0.679 | **z=8.51; p<0.001** | **z=4.89; p<0.001** |
| APOE4 | **z=9.47; p<0.001** | z=-1.05; p=0.293 | **z=9.46; p<0.001** | **z=3.22; p=0.001** |
| *Walking Latent Growth Curve Modeling* | | | | |
| *Female* | | | | |
| APOE2 | z=-0.57; p=0.569 | z=-1.68; p=0.094 | z=-1.16; p=0.244 | z=-1.02; p=0.308 |
| APOE3 | z=1.66; p=0.096 | z=1.06; p=0.291 | z=0.28; p=0.782 | z=-0.86; p=0.392 |
| APOE4 | z=1.72; p=0.085 | z=0.88; p=0.378 | z=1.32; p=0.187 | z=-1.73; p=0.084 |
| *Male* | | | | |
| APOE2 | z=0.36; p=0.717 | z=0.29; p=0.771 | z=0.42; p=0.675 | z=-0.75; p=0.455 |
| APOE3 | z=0.75; p=0.454 | z=0.40; p=0.690 | z=-0.67; p=0.502 | z=-1.50; p=0.134 |
| APOE4 | z=0.78; p=0.436 | z=1.51; p=0.130 | z=1.40; p=0.161 | z=-0.89; p=0.376 |

Black participants served as the reference group. Digit Symbol Substitution Test (DSST) and Modified Mini-Mental Status Examination (3MS).
