# Supplementary material for: Walking to protect against cognitive decline: the role of APOE genotype and sex
Source: Biol Sex Differ. 2026 Feb 21;17:58. doi: 10.1186/s13293-026-00860-6 (PMC13032221; doi:10.1186/s13293-026-00860-6)
Supplement: Supplementary file 2 — Supplementary Material 2 [file 13293_2026_860_MOESM2_ESM.docx]

|  | **DSST Initial** | **DSST Slope** | **3MS Initial** | **3MS Slope** |
| --- | --- | --- | --- | --- |
| Female |  |  |  |  |
| *APOE* ε3ε3 (Reference) (n=894) |  |  |  |  |
| *APOE* ε2ε2 (n=13) | β=-4.12; p=0.114 | β=0.21; p=0.053 | β=-2.26; p=0.147 | β=0.23; p=0.183 |
| *APOE* ε2ε3 (n=192) | **β=1.49; p=0.046** | β=0.02; p=0.425 | β=0.55; p=0.215 | **β=0.14; p=0.004** |
| *APOE* ε2ε4 (n=47) | β=0.49; p=0.729 | β=-0.06; p=0.282 | β=0.92; p=0.272 | β=-0.04; p=0.643 |
| *APOE* ε3ε4 (n=401) | β=-0.30; p=0.599 | **β=-0.11; p=<0.001** | **β=-0.78; p=0.021** | **β=-0.12; p=0.001** |
| *APOE* ε4ε4 (n=37) | β=-2.62; p=0.095 | β=-0.08; p=0.236 | **β=-3.31; p=<0.001** | **β=-0.32; p=0.002** |
| Male |  |  |  |  |
| *APOE* ε3ε3 (Reference) (n=846) |  |  |  |  |
| *APOE* ε2ε2 (n=14) | β=-1.83; p=0.467 | β=0.15; p=0.140 | β=-0.22; p=0.886 | β=0.09; p=0.563 |
| *APOE* ε2ε3 (n=219) | β=-0.23; p=0.744 | β=0.03; p=0.245 | β=0.22; p=0.605 | β=0.05; p=0.251 |
| *APOE* ε2ε4 (n=43) | β=-2.09; p=0.154 | **β=-0.12; p=0.048** | β=0.87; p=0.319 | β=-0.12; p=0.220 |
| *APOE* ε3ε4 (n=339) | β=-0.98; p=0.102 | **β=-0.06; p=0.019** | **β=-1.53; p=<0.001** | **β=-0.22; p=<0.001** |
| *APOE* ε4ε4 (n=30) | β=-2.82; p=0.103 | **β=-0.18; p=0.014** | **β=-3.37; p=0.001** | **β=-0.28; p=0.015** |
| Race (Reference: Black) | **β=8.98; p=<0.001** | β=-0.00; p=0.756 | **β=4.43; p=<0.001** | **β=0.22; p=<0.001** |
| Site (Reference: Memphis) | **β=3.37; p=<0.001** | β=0.02; p=0.087 | **β=1.40; p=<0.001** | **β=0.29; p=<0.001** |
| Age | **β=-0.82; p=<0.001** | **β=-0.01; p=<0.001** | **β=-0.32; p=<0.001** | **β=-0.04; p=<0.001** |
| Education |  |  |  |  |
| Completed High School (Reference) |  |  |  |  |
| Did not Complete High School | **β=-7.69; p=<0.001** | β=0.01; p=0.625 | **β=-4.23; p=<0.001** | **β=-0.16; p=<0.001** |
| Greater than High School | **β=4.48; p=<0.001** | β=0.02; p=0.178 | **β=2.58; p=<0.001** | **β=0.14; p=<0.001** |
| Health Score Composite | **β=-1.47; p=<0.001** | **β=-0.02; p=0.004** | **β=-0.32; p=0.009** | **β=-0.03; p=0.009** |

**Supplemental Table 1**: Full outputs for the linear models stratified by sex examining APOE genotypes differences in cognitive outcomes.

Digit Symbol Substitution Test (DSST), Modified Mini-Mental Status Examination (3MS), and Body Mass Index (BMI).
