# Supplementary material for: Walking to protect against cognitive decline: the role of APOE genotype and sex
Source: Biol Sex Differ. 2026 Feb 21;17:58. doi: 10.1186/s13293-026-00860-6 (PMC13032221; doi:10.1186/s13293-026-00860-6)
Supplement: Supplementary file 3 — Supplementary Material 3 [file 13293_2026_860_MOESM3_ESM.docx]

**Supplemental Table 2**: Z-score comparisons between males and females from the sex-stratified linear regression and latent growth curve models to quantify sex differences in APOE genotypes associations with cognitive outcomes.

|  | **DSST Initial** | **DSST Slope** | **3MS Initial** | **3MS Slope** |
| --- | --- | --- | --- | --- |
| *Main Regression Analysis* | | | | |
| APOE2 | **z=4.76; p<0.001** | z=0.40; p=0.687 | **z=1.98; p=0.048** | z=0.91; p=0.361 |
| APOE3 | **z=6.11; p<0.001** | z=1.05; p=0.292 | **z=3.23; p=0.001** | z=-1.23; p=0.219 |
| APOE4 | **z=5.16; p<0.001** | z=-0.52; p=0.601 | **z=3.94; p<0.001** | z=1.21; p=0.226 |
| *Walking Latent Growth Curve Modeling* | | | | |
| APOE2 | z=1.12; p=0.264 | z=0.97; p=0.331 | z=0.66; p=0.509 | z=-0.39; p=0.694 |
| APOE3 | z=0.87; p=0.382 | z=0.78; p=0.436 | z=0.92; p=0.355 | z=-0.29; p=0.775 |
| APOE4 | z=1.44; p=0.149 | z=1.04; p=0.298 | z=1.49; p=0.136 | z=-1.03; p=0.303 |

Males served as the reference group. Digit Symbol Substitution Test (DSST) and Modified Mini-Mental Status Examination (3MS).
