# Supplementary material for: Walking to protect against cognitive decline: the role of APOE genotype and sex
Source: Biol Sex Differ. 2026 Feb 21;17:58. doi: 10.1186/s13293-026-00860-6 (PMC13032221; doi:10.1186/s13293-026-00860-6)
Supplement: Supplementary file 4 — Supplementary Material 4 [file 13293_2026_860_MOESM4_ESM.docx]

**Supplemental Table 3**: Z-score comparisons between black and white participants from the sex-stratified linear regression and latent growth curve models to quantify race differences in APOE genotypes associations with cognitive outcomes.

|  | **DSST Initial** | **DSST Slope** | **3MS Initial** | **3MS Slope** |
| --- | --- | --- | --- | --- |
| *Main Regression Analysis* | | | | |
| *Female* | | | | |
| APOE2 | **z=5.16; p<0.001** | z=0.97; p=0.334 | **z=4.47; p<0.001** | **z=3.15; p=0.002** |
| APOE3 | **z=13.92; p<0.001** | z=-0.82; p=0.411 | **z=10.89; p<0.001** | **z=5.56; p<0.001** |
| APOE4 | **z=10.22; p<0.001** | z=0.16; p=0.874 | **z=8.79; p<0.001** | **z=3.58; p<0.001** |
| *Male* | | | | |
| APOE2 | **z=7.23; p<0.001** | z=0.86; p=0.390 | **z=6.80; p<0.001** | **z=3.04; p=0.002** |
| APOE3 | **z=12.87; p<0.001** | z=-0.41; p=0.679 | **z=8.51; p<0.001** | **z=4.89; p<0.001** |
| APOE4 | **z=9.47; p<0.001** | z=-1.05; p=0.293 | **z=9.46; p<0.001** | **z=3.22; p=0.001** |
| *Walking Latent Growth Curve Modeling* | | | | |
| *Female* | | | | |
| APOE2 | z=-0.57; p=0.569 | z=-1.68; p=0.094 | z=-1.16; p=0.244 | z=-1.02; p=0.308 |
| APOE3 | z=1.66; p=0.096 | z=1.06; p=0.291 | z=0.28; p=0.782 | z=-0.86; p=0.392 |
| APOE4 | z=1.72; p=0.085 | z=0.88; p=0.378 | z=1.32; p=0.187 | z=-1.73; p=0.084 |
| *Male* | | | | |
| APOE2 | z=0.36; p=0.717 | z=0.29; p=0.771 | z=0.42; p=0.675 | z=-0.75; p=0.455 |
| APOE3 | z=0.75; p=0.454 | z=0.40; p=0.690 | z=-0.67; p=0.502 | z=-1.50; p=0.134 |
| APOE4 | z=0.78; p=0.436 | z=1.51; p=0.130 | z=1.40; p=0.161 | z=-0.89; p=0.376 |

Black participants served as the reference group. Digit Symbol Substitution Test (DSST) and Modified Mini-Mental Status Examination (3MS).
